# Short Tandem Repeat 3D Structure Database (STR3SD): A Resource for Structural Biology Research of Short Tandem Repeats in Neurodegenerative Disorders

**DOI:** 10.3390/ijms27156872

**Published:** 2026-07-31

**Authors:** Kaitengjie Jie, Anqi Song, Yang Wang, Yu Liu, Yang Liu, Menghao Guo, Zhiming Zhang, Ning Xu, Yi Tao, Liqi Wan, Jiezhong Qiu, Pei Guo

**Affiliations:** 1 College of Pharmaceutical Science, Zhejiang University of Technology, Hangzhou 310014, Chinaxuning@zjut.edu.cn (N.X.);; 2 Hangzhou Institute of Medicine (HIM), Chinese Academy of Sciences, Hangzhou 310022, China; wyang5good@163.com (Y.W.);; 3 College of Materials Science and Engineering, Zhejiang University of Technology, Hangzhou 310014, China; 4 Zhejiang Lab, Hangzhou 311100, China

**Keywords:** short tandem repeats, neurodegenerative diseases, 3D structure, database, structural polymorphism, drug design, nucleic acid complexes

## Abstract

The Short Tandem Repeat 3D Structure Database (STR3SD) is a web-based database that provides a comprehensive resource for structural biology research of short tandem repeats (STRs) in cancers and neurodegenerative diseases. STR3SD contains three-dimensional (3D) structures of STRs surveyed from literature. The data are organized into three main categories, including 3D structures of nucleic acids only, nucleic acids–protein complexes, and nucleic acids–ligand complexes. Under these categories, each entry is annotated with repeat type, molecular type (DNA or RNA), sequence, PDB ID, structural component, ligand name, structural determination method, experimental conditions (temperature, pH, and ion), PubMed ID, and interactive 3D structure view. The database is built on direct literature investigation by human experts and serves as a crucial tool for studying structures and functions of STRs in cancers and neurodegenerative diseases, supporting research on structural polymorphisms and pathogenic mechanisms of STRs, and facilitating drug design targeting STRs for disease therapy.

## 1. Introduction

Short tandem repeats (STRs) are repetitive DNA elements composed of tandem arrays of 1–6 base pairs [1]. STRs are widely distributed in exons, introns and regulatory regions, comprising nearly 7% of the human genome [2]. STRs exhibit a high propensity for genetic instability, whereby the number of repeats increases or decreases dynamically, namely repeat expansions or contractions. Such genetic instability arises from error-prone processes during DNA replication, repair, recombination, and transcription [3,4], and is often mediated by unusual structures of STRs that promote repeat-length changes [5]. In particular, STR expansions have been reported to associate with more than 50 human neurodegenerative diseases [6], such as Huntington’s disease (HD), spinocerebellar ataxias (SCAs), myotonic dystrophies (DMs), frontotemporal dementia (FTD) and amyotrophic lateral sclerosis (ALS). The threshold number of repeats that cause disease onset varies among different types of STRs in different diseases. For instance, the ranges of CAG repeats in the *HTT* gene of normal individuals and HD patients are 9–29 and 36–121, respectively [7], whereas the ranges of GGGGCC repeats in the *C9orf72* gene of normal individuals and ALS/FTD patients are 2–19 and 250–1600, respectively [8,9]. Recently, Hujoel et al. have analyzed DNA sequencing data from more than 900,000 biobank participants and revealed widespread germline variation and somatic repeat instability [10], and Erwin et al. analyzed 2622 cancer genomes from 2509 patients and identified recurrent repeat expansions in human cancer genomes [11]. These findings position STRs as both common sources of heritable and somatic variation and as consequential contributors to human diseases, particularly in neurodegenerative disorders and cancers.

The pathogenicity of STRs is shaped not only by repeat length but also by their secondary and three-dimensional (3D) structures. While the canonical right-handed double-helical DNA structure (B-DNA) is well known to be responsible for maintaining genomic and hereditary stability [12], STRs display significant structural diversity due to the unique nature of their sequences [13]. STRs with distinct sequence compositions and varying numbers of repeat units can fold into a wide variety of non-canonical DNA and RNA structures, such as hairpins formed by CAG and CUG repeats [14,15], Z-DNA by CG repeats [16], triplexes or H-DNA by GAA repeats [17], G-quadruplexes and hairpins by GGGGCC repeats [18,19], and i-motifs by TCCCCC repeats [20]. The non-B DNA structures formed by STRs affect cellular processes of DNA replication, transcription, repair and recombination [21,22]. They not only serve as structural intermediates that drive repeat expansions [23], but also act as roadblocks that impede polymerase progression and inhibit gene expression [24]. At the RNA molecular level, expanded RNA repeats exhibit toxic gain-of-function. They adopt unusual structures that sequester various RNA-binding proteins (RBPs) to form nuclear RNA foci [25,26]. Sequestration of RBPs that usually function in RNA splicing and metabolic processes will lead to splicing errors and subsequent perturbations of cellular functions.

As STRs are associated with various diseases, therapeutic strategies targeting their 3D DNA and RNA structures have garnered increasing interest. For example, a naphthyridine-azaquinolone ligand was designed to selectively bind A·A mismatches within CAG repeats [27], and triggered contractions of CAG/CTG repeats in cells derived from HD patients [28]. In addition, a conjugate molecule that combines an RNA-binding domain (triaminotriazine-acridine) with a synthetic mimic of ribonuclease A was capable of binding and cleaving pathogenic expanded CUG repeats in a *Drosophila* model of DM1 [29]. Despite these advances, general principles for designing small molecules that target 3D structures of STRs remain elusive [30,31], with current knowledge largely restricted to a few case-specific insights derived from structure-guided studies. Hence, there is an urgent need to compile a comprehensive 3D structural database of STRs, which would enable computational and artificial intelligence-based approaches for the design of therapeutics targeting these repetitive elements.

Given the biological significance of STRs, several databases focusing on STRs have been developed, such as the STRBase [32], ATCC STR Database [33], and STRipy [34]. STRBase provides forensic STR marker information for law enforcement researchers, ATCC STR Database offers cell line authentication for cell biology researchers, and STRipy enables pathogenic STR detection for clinical geneticists. Despite the genetic and clinical insights provided by these databases, there is currently no database that specifically collects high-resolution 3D structural information of STRs, encompassing both experimentally determined structures by X-ray crystallography, nuclear magnetic resonance (NMR) spectroscopy, and cryogenic electron microscopy (cryo-EM), as well as computational models by molecular dynamics (MD) simulations. Furthermore, the 3D structures of STRs deposited in the Protein Data Bank (PDB) lack complete documentation of experimental conditions (e.g., ion concentration, temperature, and pH). In addition, MD simulations of STR structures and MD simulation-based virtual screening for STR-targeting drugs have not been systematically curated or subjected to integrated analysis [35].

Herein, we report a Short Tandem Repeat 3D Structure Database (STR3SD) that comprehensively archives 3D structural data for STRs, STR–protein, and STR–ligand complexes. This database allows data retrieval based on sequence, structural method, and other metadata, offers flexible data download options, and provides operable 3D molecular visualization. STR3SD will facilitate researchers from various fields, including structural biology, neuroscience, chemistry and pharmaceutics, in their studies on STRs and the relevant human diseases.

## 2. Results

### 2.1. Overview of STR3SD

**Figure 1:**
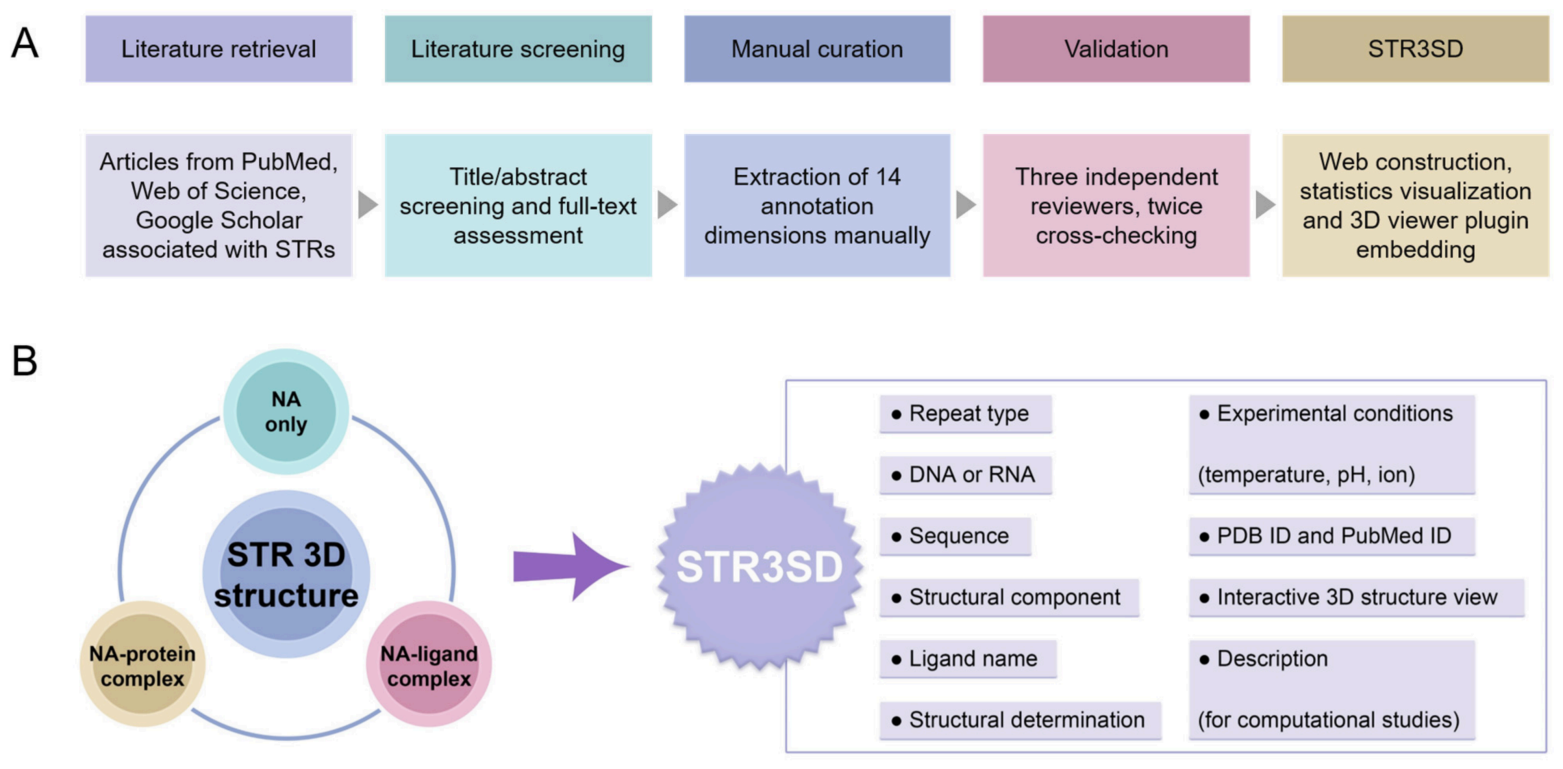
Construction and attributes of STR3SD: (**A**) Workflow of database construction. (**B**) Attributes of STR3SD. The central circle represents the 3D structures of STRs, which are classified into three data subsets shown as colored satellites: NA only (green), NA–ligand complexes (magenta) and NA–protein complexes (olive). The right panel lists the annotation dimensions of each entry, including Repeat type, DNA or RNA, Sequence, Structural component, Ligand name, Structural determination, Experimental conditions (temperature, pH, and ion), PDB ID and PubMed ID, Interactive 3D structure view and Description for computational studies. NA, nucleic acid.

To establish STR3SD, we systematically searched PubMed, Web of Science, and Google Scholar for publications pertaining to STRs and 3D structures by human experts. This search retrieved 5483 articles published up to 1 June 2026. Through successive rounds of title/abstract screening, full-text assessment, and manual curation against predefined inclusion criteria that required the presence of experimentally determined or computationally modeled 3D structures of STRs, 162 qualified entries were ultimately retained. All the retained data underwent independent cross-checking by three trained reviewers to ensure accuracy and consistency. The workflow for data extraction and curation is illustrated in Figure 1A.

Each entry in STR3SD represents a single curated 3D structure record. For experimental studies, one entry corresponds to one PDB record. For computational studies, one entry corresponds to one MD simulation structure or simulation-based virtual screening. When an article reports multiple PDB records or multiple independent simulations, each of them is registered as an independent entry, and these entries share the same PubMed ID. Entries in STR3SD are annotated across the following fields. The field of “Repeats” means the sequence of the repetitive unit of STRs. The field of “DNA or RNA” means the type of nucleic acid. The field of “Sequence” means the nucleotide sequence in single-letter format with a 5′-to-3′ direction. The field of “PDB ID” means the accession code of the structure in the PDB. The field of “Ligand name” means the name of the bound molecule. The field of “Method” means the approach used to obtain the structure. The field of “PubMed ID” means the identifier of the source article in PubMed. The field of “Temperature” means the temperature of the crystallization for X-ray structures or the sample temperature during NMR spectroscopy. The field of “Ion” means the ionic composition of the crystallization or NMR buffer. The field of “pH” means the pH value of the crystallization buffer or NMR sample. The field of “3D structure” directs to an integrated 3D structure viewer [36,37] of the determined structure in NMR and X-ray studies, or the initial structure in MD studies. The field of “Description” shows a professional summary of the main conclusion for MD simulations and virtual screenings. The symbol “-“ indicates that the corresponding information was not reported in the original article.

To distinguish the type of ligand bound to the structure of STRs, we introduced a “Data subset” dimension to categorize whether the STRs are bound to a ligand or protein. Three categories are presented, including structures of nucleic acids only (NA only), nucleic acid–ligand complexes (NA–ligand), and nucleic acid–protein complexes (NA–protein). An additional “Component” dimension was introduced to further refine the molecular composition within each category, including DNA only, RNA only, DNA–ligand complex, RNA–ligand complex, DNA–protein complex, RNA–protein complex, RNA-PNA complex and DNA/RNA hybrid. In other words, the “Data subset” dimension classifies each entry according to the presence and type of the bound partner, whereas the “Component” dimension describes the detailed composition of the structure itself. Figure 1B shows the attributes of STR3SD.

### 2.2. Data Curation Reveals the Structural Diversity and Research Focus of STRs

**Figure 2:**
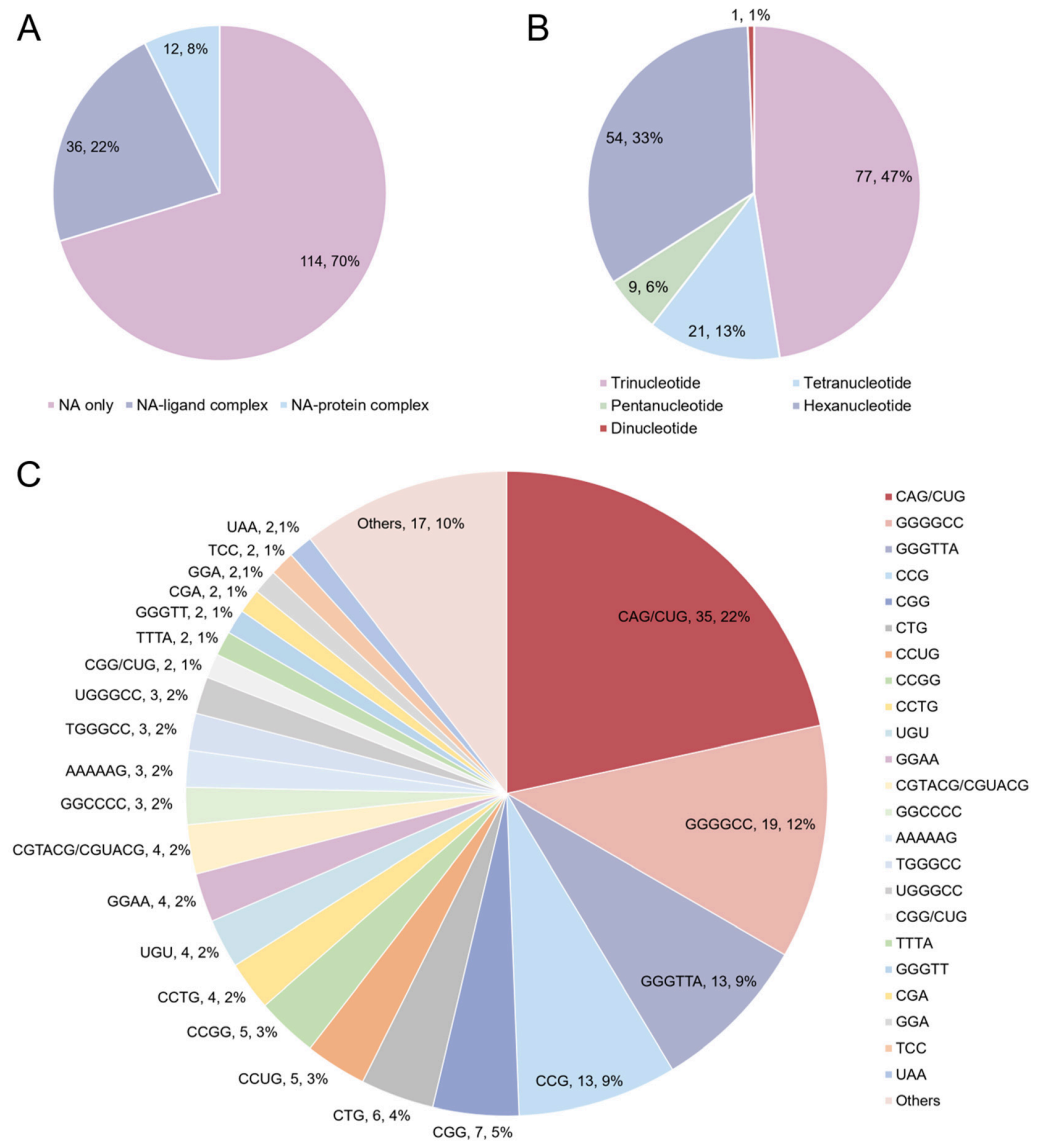
The statistics of STR3SD: (**A**) Three data subsets including NA only, NA–ligand complexes and NA–protein complexes. (**B**) Repetitive units with different lengths. (**C**) Different repetitive units.

The statistics of data subsets and repeat lengths are displayed in Figure 2A,B. Research on 3D structures predominantly focuses on nucleic acids without bound ligands or proteins (NA only, 70%). Among these, trinucleotide repeats (47%) and hexanucleotide repeats (33%) are the most extensively studied. These statistical charts are automatically generated on the website homepage. Figure 2C illustrates the distribution of different repetitive units, with CAG/CUG (22%) and GGGGCC (12%) repeats being the most frequently studied, reflecting the predominant trends in current research. All the percentages are calculated based on the number of entries.

### 2.3. Interactive Web Interface for Data Retrieval, Visualization, and Export

#### 2.3.1. Web Interface

STR3SD is a freely accessible, web-based online database available at https://str3sd.fnadb.com. It features a user-friendly graphical interface, offering multi-button search functionalities that present results in categorized order. The homepage is organized into five main sections: database introduction, general search, advanced search, data statistics, and update log.

#### 2.3.2. Search Section

**Figure 3:**
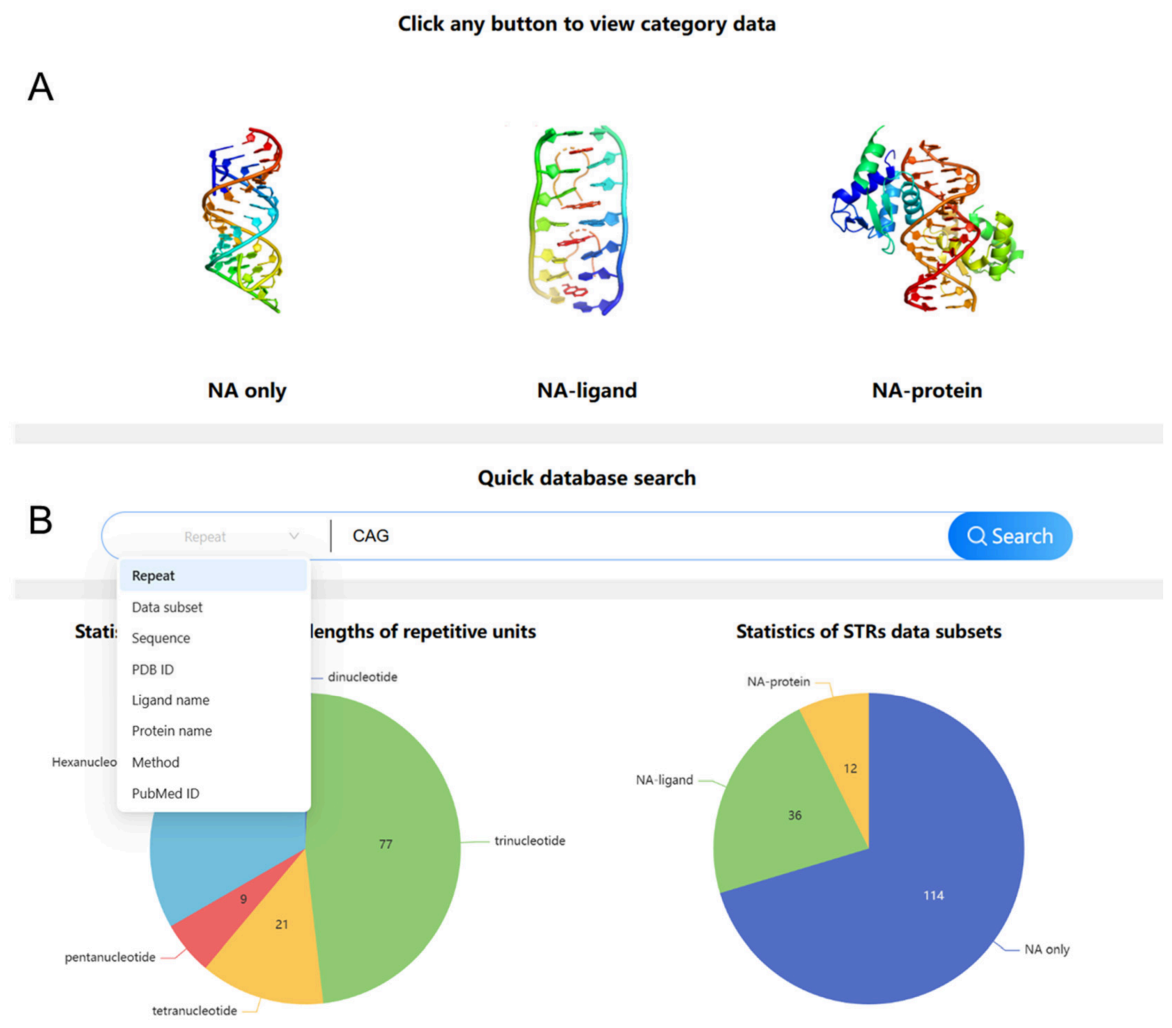
The search module of STR3SD. (**A**) General search. Three category icons are provided, representing “NA only” (STRs without bound partners), “NA–ligand” (nucleic acid–ligand complexes) and “NA–protein” (nucleic acid–protein complexes). Clicking an icon directly returns all entries of the corresponding category. (**B**) Advanced search. Eight query domains (Repeat, Data subset, Sequence, PDB ID, Ligand name, Protein name, Method and PubMed ID) are available in the dropdown menu for precise retrieval.

STR3SD provides two search sections for user access: general search and advanced search. In the general search module, three main categories including “NA only”, “NA–ligand” and “NA–protein” are presented (Figure 3A). The “NA only” category collects 3D structures of STRs without any bound partner, the “NA–ligand” category collects structures of STRs in complex with small molecule ligands, and the “NA–protein” category collects structures of STRs bound to proteins. Clicking on the category icon displays search results for a broad class of STRs, and thus the general search is designed for one-click browsing of an entire main category. In the advanced search module, the dropdown menu allows users to search for results by typing keywords for eight domains including Repeat, Data subset, Sequence, PDB ID, Ligand name, Protein name, Method and PubMed ID (Figure 3B), which enables precise, field-specific retrieval. Switching between any advanced search fields dynamically displays sample entries. These flexible search modules assist researchers in efficiently exploring the database and achieving an optimized search experience.

#### 2.3.3. Result Section

**Figure 4:**
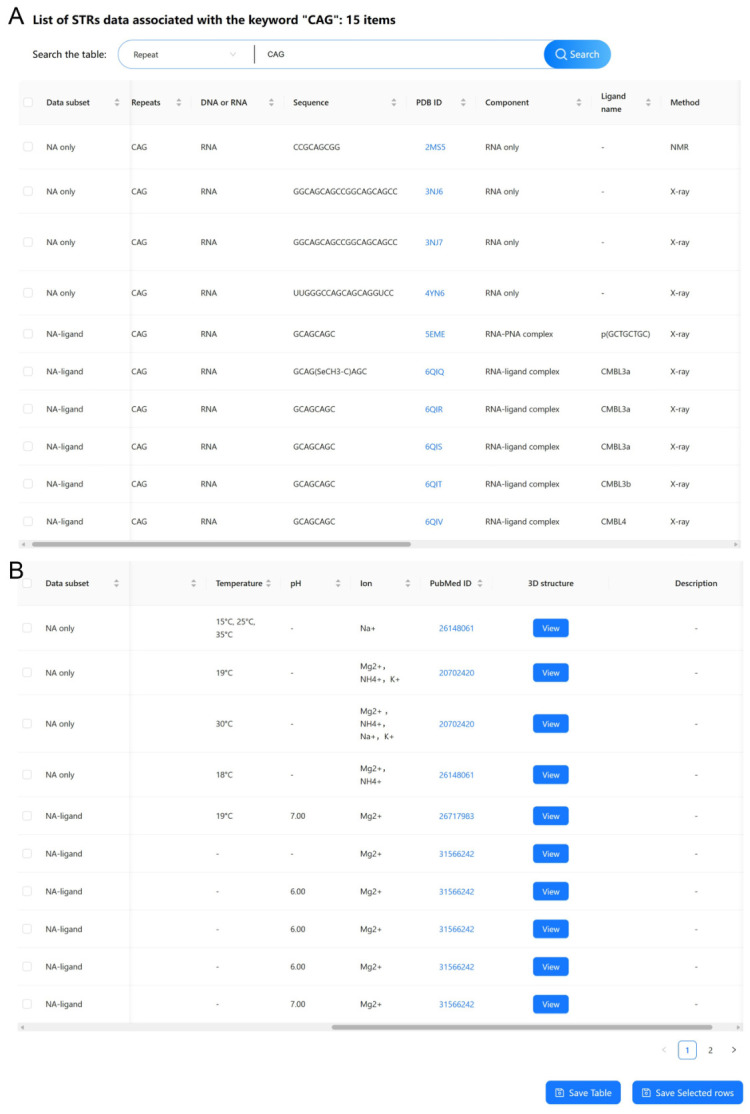
Screenshot of the STR3SD database search results using “CAG” as the query keyword (15 entries with CAG as the repeat unit are retrieved): (**A**) Structural feature and method columns, including Data subset, Repeats, DNA or RNA, Sequence, PDB ID, Component, Ligand name and Method. (**B**) Experimental condition and interactive function columns, including Temperature, pH, Ion, PubMed ID, 3D structure and Description. Hyperlinked PDB IDs and PubMed accession numbers (in blue) direct users to the corresponding PDB and PubMed webpages, respectively, and the blue “View” buttons launch the integrated 3D structure visualization tool. For experimentally determined structures, the Description field is designated as “-”.

The results are presented on a results page in a tabular format with one record per row and multiple data columns (Figure 4). As an example, searching “CAG” in the Repeat field of the advanced search module returns 15 entries (Figure 4). These entries cover RNA-only structures as well as RNA–ligand, RNA-PNA and DNA–ligand complexes, encompassing annotated sequences, structural determination methods, ligand names, experimental conditions (temperature, pH, and ion composition), and hyperlinked PDB IDs, PubMed IDs, and interactive 3D structure views. For example, the first entry (PDB ID: 2MS5) corresponds to an RNA containing one CAG repeat as determined by NMR spectroscopy at 15, 25, and 35 °C in the presence of Na^+^. The Ligand name of “-“ means the absence of a bound partner. The pH field is marked as “-“, indicating that the pH condition was not reported in the original publication. The database provides multiple interactive functions. The hyperlinked PDB ID (in blue) directs users to the corresponding PDB entry. The hyperlinked PubMed accession number (in blue) links to the PubMed abstract page. The “View” button under the “3D structure” column launches the online 3D structure viewer. The Description field is marked as “-“ for this entry, as it is an experimentally determined structure.

**Figure 5:**
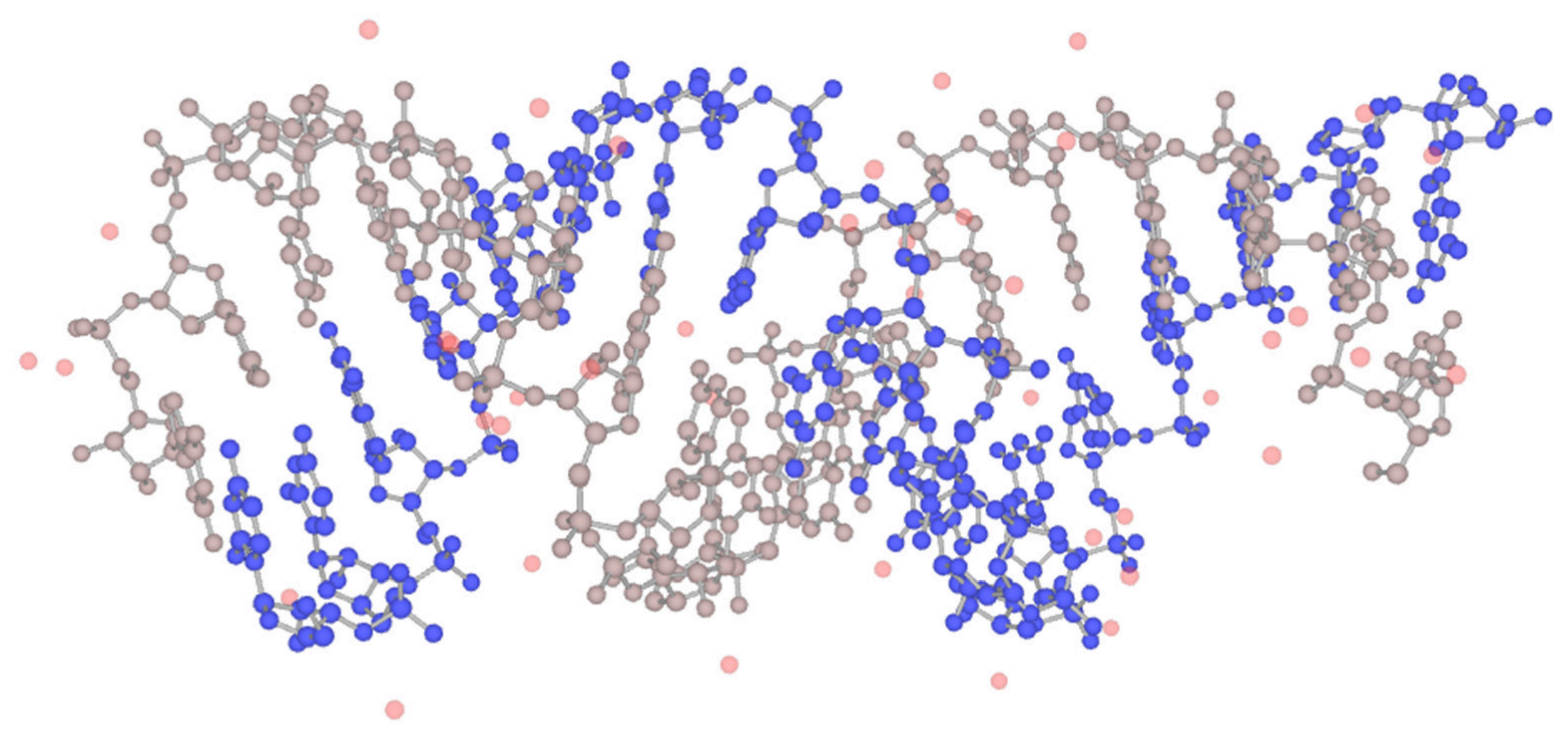
The online 3D visualization function of STR3SD by the embedded LiteMol viewer, as exemplified by the CUG-repeat RNA structure (PDB ID: 1ZEV) [38]. The structure is shown as a ball-and-stick model, with the two RNA strands depicted as gray and blue spheres, respectively, and ordered water molecules in the crystal lattice shown as pink spheres.

Notably, the LiteMol plugin is embedded to enable online viewing and interactive manipulation of 3D structures [36,37]. Users can click the “View” button to visualize a 3D structure and switch the rendering mode between ball-and-stick, surface and van der Waals sphere models (Figure 5). All or selected data can be exported directly from the search results page as an .xlsx file. Using various search combinations, users can sort the literature and obtain statistical summaries at the database level.

### 2.4. Meta-Analysis of Structural Polymorphisms Across Disease-Associated STRs

**Figure 6:**
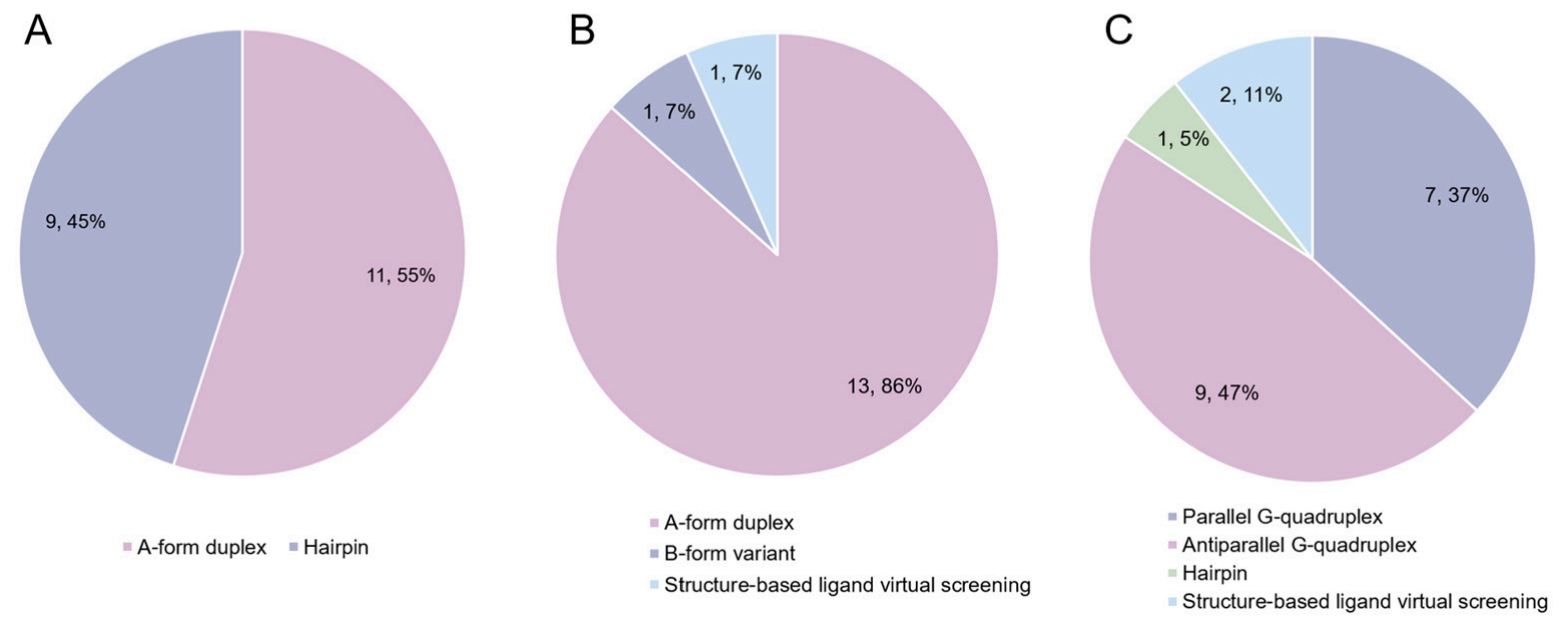
The structural polymorphisms of the top 3 STRs: (**A**) CUG repeats. (**B**) CAG RNA repeats. (**C**) GGGGCC DNA and RNA repeats.

STR3SD enables systematic meta-analysis of the structural polymorphism of STRs efficiently. As shown in Figure 6, we utilized STR3SD to integrate and visually summarize the three most common types of STR structural polymorphisms. For CUG repeats associated with DM1 [39], nearly all the existing studies focus on their A-form RNA duplex structure, with approximately 45% of these studies further investigating their hairpin structure. Regarding CAG repeats associated with HD and many SCAs [40], the vast majority of studies (86%) concentrate on the A-form RNA duplex, while only 7% of structural research involves the B-form variant. In addition, structure-based virtual screening has identified the small molecule somniferine, which exhibits high-affinity binding to CAG repeats. In the case of GGGGCC repeats associated with ALS/FTD [41], most studies focus on the antiparallel G-quadruplex (47%), followed by the parallel G-quadruplex (37%). A small proportion of studies (5%) report that this sequence can also form a hairpin structure. In terms of drug discovery, virtual screening results integrated by STR3SD suggest that the small molecules Au(TMX)_2_ and orotate are two reported potential ligands targeting the 3D structures of GGGGCC repeats [42,43]. All the percentages are calculated based on the number of entries.

In sum, the powerful data mining and pattern recognition capabilities of STR3SD allow it to not only present individual data points but also greatly facilitate researchers in performing cross-study comparisons and meta-analyses. This helps reveal structural preferences, research trends, and even underexplored areas associated with specific STR sequences, such as GGGGCC repeats, across different studies.

### 2.5. Advantages and the Expanding Scope of STR3SD

To the best of our knowledge, currently there is no other database specifically dedicated to the study of 3D structures of STRs. While researchers can query structural data through databases such as PDB, the information retrieved may be incomplete or even contain errors. STR3SD offers meta-analysis capabilities for homologous STRs, providing more accurate experimental conditions, an interactive interface for 3D structure visualization, and multi-level data categorization. This demonstrates that STR3SD delivers a more precise, goal-oriented, and user-friendly experience, and enables seamless comparisons across different studies. STR3SD is updated every 3 months to incorporate the latest research, thereby surpassing the limitations of static reviews. Additionally, to facilitate access to original articles and structural data, PubMed IDs and PDB IDs are clearly listed.

## 3. Discussion

STRs play important biological roles, with their sequences tuning gene expression [13] and their expansions driving more than 50 human diseases [6]. Because the pathogenicity of STRs is intimately linked to the non-canonical 3D structures they adopt, a database dedicated to the 3D structures of STRs is of great significance for elucidating disease mechanisms and developing STR-targeting drugs. Over the past two decades, a number of STR-related databases have been developed (Table 1). For disease and phenotype-related databases, STRipy [34] enables the detection of pathogenic STRs from sequencing data. In forensic and human identity testing, STRBase [32] provides STR marker information, and STRidER [44] offers quality-controlled STR allele frequencies. In the field of cell line authentication, ATCC STR Database [33] supports the verification of cell identity. For genome-wide STR identification and population variation analysis, TRDB [45], WebSTR [46], SNPSTR [47], UgMicroSat *db* [48] and Microsatellites Explorer [49] catalog the sequences, genomic coordinates and population variation in STRs identified by computational mining of genomes. Despite the wealth of genomic, forensic and clinical information and dedicated search algorithms, none of them provide high-resolution structure information of STRs.

In terms of nucleic acid structural databases, NAKB [50] archives all experimentally determined nucleic acid 3D structures held by the NDB and PDB, and ONQUADRO [36] specializes in experimentally determined quadruplex structures with detailed geometric annotations. These databases are not specific to STRs, and they do not document experimental conditions (e.g., ion concentration, temperature, pH) or computational studies such as MD simulations and MD simulation-based virtual screening.

To facilitate the development of small-molecule drugs targeting 3D structures of pathogenic STRs, our STR3SD represents the first database collecting the experimentally determined high-resolution 3D structures of STRs. Because STR3SD focuses on well-defined, disease-associated STRs for which 3D structures have been experimentally determined or computationally modeled, no STR searching algorithm is required. The unique contribution of STR3SD lies in the systematic manual integration, cross-validation and functional annotation of dispersed structural data, which transforms isolated literature results into a unified and accessible resource that facilitates structure-based drug design targeting STRs.

By providing standardized 3D structural data and comprehensive annotations, STR3SD lays a foundation for investigating the “sequence-structure-function” relationships in STRs. It facilitates the exploration of structural polymorphisms in STRs, aids in identifying disease-associated conformations, and enables computational drug design and modeling targeting STR ligands. The database supports searches based on sequence, structural attributes, and other metadata, offers flexible data export options, and includes an interactive 3D molecular visualization interface. STR3SD represents a much-needed and valuable resource in the fields of nucleic acid structural biology and precision medicine.

STR3SD meticulously documents nearly all the available STR 3D structures, supports large-scale data analysis, assists researchers in optimizing experimental design, and provides pharmaceutical companies with targetable sequences to accelerate drug discovery. For academic and research institutions, it offers a new perspective on STR research progress. The author team is committed to maintaining and updating STR3SD regularly, addressing issues, and expanding features to enhance user experience. We cordially invite users to submit error reports, seek assistance, or suggest new functionalities via email or other contact channels.

## 4. Materials and Methods

### 4.1. Data Collection

We first systematically collected, reviewed, and categorized a vast amount of information related to various attributes of STRs. By searching articles from PubMed, Web of Science and Google Scholar published up to 1 June 2026 using keywords for “short tandem repeats” and “3D structure”, 5483 articles were obtained for further screening. Subsequently, refined STR-related keywords such as disease names of “Huntington’s Disease” or “Fragile X Syndrome”, and STR types of “CAG repeats” or “GGGGCC repeats” in titles/abstracts were used to further identify literature specifically related to 3D structures of STRs, followed by full-text assessment to confirm the presence of experimentally determined or computationally modeled 3D structures of STRs, which were then subjected to the next stage of data extraction.

The extracted data were systematically organized into the following fields, with definitions and sources specified. The field of “Data subset” means the main category classified by the binding partner, which is annotated by the curator based on the original article. The field of “Repeats” means the sequences of the repetitive unit, which is sourced from the original article. The field of “DNA or RNA” means the type of nucleic acid, which is sourced from the original article. The field of “Sequence” means the nucleotide sequence in single-letter format with a 5′-to-3′ direction, which is sourced from the original article. The field of “PDB ID” means the accession code of the structure in the PDB, which is sourced from the original article and cross-validated against the PDB. The field of “Component” means the fine classification of the molecular composition, which is annotated by the curator based on the original article. The field of “Ligand name” means the name of the bound molecule, which is sourced from the original article. The field of “Method” means the approach used to obtain the structure, which is sourced from the original article. The field of “Temperature” means the temperature of crystallization for X-ray structures or the sample temperature during NMR spectroscopy, which is sourced from the original article. The field of “pH” means the pH value of the crystallization or NMR buffer, which is sourced from the original article. The field of “Ion” means the ionic composition of the crystallization or NMR buffer, which is sourced from the original article. The field of “PubMed ID” means the identifier of the source article with a hyperlink to PubMed, which is annotated by the curator based on PubMed. The field of “3D structure” directs to the interactive structure view, which is sourced from the PDB and the original article. The field of “Description” displays the professional summary of the study for MD simulations and virtual screenings, which is extracted from the original article with curator interpretation.

When a single article reported multiple PDB records or multiple independent computational models, each PDB record or computational model was registered as an independent entry, with all the entries from the same article sharing the same PubMed ID.

### 4.2. Data Validation

All the data extractors received standardized procedural training to ensure consistency in their operations. The data from each article were independently reviewed and consolidated by three well-trained reviewers. After all the data entries were completed, random sampling was performed twice for verification against the original sources to ensure the accuracy and reliability of the final dataset. All the 3D structures accessible in STR3SD were subjected to online rendering tests to ensure accurate and rapid interactive visualization.

### 4.3. Database Architecture and Implementation

STR3SD is built on a modern full-stack architecture that emphasizes modularity, maintainability, and a seamless user experience. The system strictly separates the backend service layer from the frontend presentation layer.

The server-side application is developed using the RuoYi framework (v3.8.1, RuoYi Open Source Project, Zhanjiang, Guangdong, China), a rapid development platform based on Spring Boot (v2.5.8, Broadcom Inc., San Jose, CA, USA), Spring Security (v5.5.4, Broadcom Inc., San Jose, CA, USA), and MyBatis-Plus (v3.4.2, baomidou Open Source Project, China). It employs a microservice architecture managed by Spring Cloud Alibaba for scalability and resilience. Core services are registered and configured through Nacos (v2.2.1, Alibaba Group, Hangzhou, Zhejiang, China). Data are persisted in a MySQL database (v5.7.40, Oracle Corporation, Redwood City, CA, USA), with Redis (v6.1.5, Redis Ltd., Mountain View, CA, USA) utilized for high-performance caching. This backend stack provides a robust, secure, and efficient foundation for data management, API services, and business logic. The interactive web interface is constructed as a single-page application (SPA) using React (v19.1.0, Meta Platforms, Inc., Menlo Park, CA, USA), with Ant Design (v6.2.0, Ant Group, Hangzhou, Zhejiang, China) as the core user interface (UI) component library. This combination enables the development of a responsive, dynamic, and visually consistent user interface that works flawlessly across desktop and mobile platforms. Specialized functionalities are integrated into this core architecture. The interactive 3D molecular viewer is implemented by embedding the LiteMol plugin (v2, CEITEC-Masaryk University, Brno, Czech Republic) within the React application. Data exploration is enhanced through dynamic table components and advanced search filters. Users can conveniently export query results as .xlsx files for offline analysis.

## Figures and Tables

**Table 1:** Comparison of STR-related databases.

Database	Description	Reference
STRipy	Genotyping of pathogenic STRs from sequencing data for clinical diagnosis	[34]
STRBase	STR marker information for forensic human identity testing	[32]
STRidER	Quality-controlled autosomal STR allele frequency database for forensic genetics	[44]
ATCC STR Database	STR profiles for human cell line authentication	[33]
TRDB	Tandem repeat repository with analysis tools for genomic DNA	[45]
WebSTR	Population-wide STR variation in humans	[46]
SNPSTR	Compound microsatellite-SNP markers in human and model organisms	[47]
UgMicroSat *db*	Microsatellites mined from UniGene sequences of 80 genomes	[48]
Microsatellites Explorer	STRs across 117,253 organisms, T2T genomes and human pangenome haplotypes	[49]
STR3SD	3D structures of all STRs, with experimental conditions, MD simulations and virtual screening records	This work

## Data Availability

The STR3SD database will be updated regularly (every 3 months). It is now publicly and freely available at https://str3sd.fnadb.com/. Related data will be available upon request from the authors.

## Abbreviations

The following abbreviations are used in this manuscript:

3D	Three-Dimensional
ALS	Amyotrophic Lateral Sclerosis
Cryo-EM	Cryogenic Electron Microscopy
DM	Myotonic Dystrophy
FTD	Frontotemporal Dementia
HD	Huntington’s Disease
MD	Molecular Dynamics
NA	Nucleic Acid
NMR	Nuclear Magnetic Resonance
PDB	Protein Data Bank
RBP	RNA-Binding Protein
SCA	Spinocerebellar Ataxia
SPA	Single-Page Application
STR3SD	Short Tandem Repeat 3D Structure Database
STR	Short Tandem Repeat
UI	User Interface
